# A Novel Digital Algorithm for Identifying Liver Steatosis Using Smartphone-Captured Images

**DOI:** 10.1097/TXD.0000000000001361

**Published:** 2022-08-04

**Authors:** Katherine Xu, Siavash Raigani, Angela Shih, Sofia G. Baptista, Ivy Rosales, Nicola M. Parry, Stuti G. Shroff, Joseph Misdraji, Korkut Uygun, Heidi Yeh, Katherine Fairchild, Leigh Anne Dageforde

**Affiliations:** 1 Quest for Intelligence, College of Computing, Massachusetts Institute of Technology, Cambridge, MA.; 2 Division of Transplant Surgery, Massachusetts General Hospital, Boston, MA.; 3 Department of Pathology, Massachusetts General Hospital, Boston, MA.; 4 Center for Engineering in Medicine and Surgery, Massachusetts General Hospital, Harvard Medical School, Boston, MA.; 5 Division of Comparative Medicine, Massachusetts Institute of Technology, Cambridge, MA.; 6 Department of Pathology, Yale New Haven Hospital, New Haven, CT.

## Abstract

Access to lifesaving liver transplantation is limited by a severe organ shortage. One factor contributing to the shortage is the high rate of discard in livers with histologic steatosis. Livers with <30% macrosteatosis are generally considered safe for transplant. However, histologic assessment of steatosis by a pathologist remains subjective and is often limited by image quality. Here, we address this bottleneck by creating an automated digital algorithm for calculating histologic steatosis using only images of liver biopsy histology obtained with a smartphone.

**Methods.:**

Multiple images of frozen section liver histology slides were captured using a smartphone camera via the optical lens of a simple light microscope. Biopsy samples from 80 patients undergoing liver transplantation were included. An automated digital algorithm was designed to capture and count steatotic droplets in liver tissue while discounting areas of vascular lumen, white space, and processing artifacts. Pathologists of varying experience provided steatosis scores, and results were compared with the algorithm’s assessment. Interobserver agreement between pathologists was also assessed.

**Results.:**

Interobserver agreement between all pathologists was very low but increased with specialist training in liver pathology. A significant linear relationship was found between steatosis estimates of the algorithm compared with expert liver pathologists, though the latter had consistently higher estimates.

**Conclusions.:**

This study demonstrates proof of the concept that smartphone-captured images can be used in conjunction with a digital algorithm to measure steatosis. Integration of this technology into the transplant workflow may significantly improve organ utilization rates.

## INTRODUCTION

Liver transplantation remains the only definitive therapy for end-stage liver disease. Despite its success, timely access to this lifesaving procedure is limited by a significant organ shortage. As a result, up to 30% of waitlisted patients die before a suitable donor liver becomes available.^1^ One factor contributing to the shortage is the prevalence of hepatocellular macrosteatosis, or fatty liver, due to donor metabolic syndrome or alcohol use. The presence of histologic macrosteatosis (also called large-droplet steatosis) in hepatocytes beyond a 20% to 30% threshold has been associated with decreased graft survival, as well as increased early allograft dysfunction and postreperfusion syndrome.^2-4^ Despite reported case series of successful transplantation of livers with moderate (30%–60%) or severe (60+%) macrosteatosis^5-7^ and efforts at enhanced donor–recipient matching,^8,9^ steatotic grafts are still commonly discarded because of concern about poor graft outcomes.^10-12^ Moreover, the organ shortage is likely to be exacerbated as the prevalence of obesity, metabolic syndrome, and nonalcoholic fatty liver disease continues to increase.^13^

The subjective nature of evaluating donor liver steatosis during the course of organ procurement further contributes to the high discard rate of these grafts. Visual assessment of liver color and contour can be unreliable,^14,15^ and although liver biopsy with rapid frozen section assessment by an experienced liver pathologist has been considered the gold standard, several technical and interpretive factors directly affect its accuracy. In the latter scenario, frozen sectioning with hematoxylin and eosin (H&E) stain is often limited by the presence of processing artifacts that can mimic lipid droplets resulting in overestimation of macrosteatosis. In addition, because of the unpredictable and time-sensitive nature of organ procurement, frozen sections are often assessed by pathologists at donor hospitals without specialist training in liver pathology. As a result, there is poor interobserver agreement even when multiple liver pathologists score the same biopsy slide.^16^ To address this limitation, intense research in automating the assessment of liver steatosis has been undertaken in the last decade. Several groups have reported impressive results with the use of artificial intelligence or machine-learning (ML) algorithms trained to score the degree of steatosis using whole-slide images.^17-21^

To improve the expediency of the donor liver assessment workflow, we hypothesized that a non-ML algorithm could accurately assess liver steatosis in frozen sections using smartphone-based images taken in tandem with a simple light microscope. This would simulate the real-time assessment of liver steatosis during organ procurement with frozen section biopsy but with the added benefit of providing an automated steatosis score from user-captured smartphone images at low cost. Using a cohort of 80 transplanted livers with available histology, we created and validated an image segmentation algorithm and compared its accuracy against steatosis scores from pathologists with a range of expertise in liver pathology. Unlike artificial intelligence systems that train a model on pathologist annotations, our approach primarily relies on non-ML computer vision algorithms to detect steatotic regions, thereby avoiding interobserver discrepancies due to subjective visual estimates.

## MATERIALS AND METHODS

### Cohort Selection

An algorithm development image cohort was derived from 21 human liver biopsies with macrosteatosis ranging from 0% to 80% from a previously reported study cohort.^11^ Both formalin-fixed paraffin-embedded and separately frozen section tissue biopsies were available for this cohort. For algorithm validation, 91 H&E-stained frozen donor liver biopsies from 80 livers undergoing transplantation at Massachusetts General Hospital (MGH) between January 2014 and December 2019 were obtained from the MGH Pathology department. Macrosteatosis ranged from 0% to 30% in this cohort. The MGH Institutional Review Board approved this study (#2019P002930).

### Smartphone-Based Imaging

Tissue slide images were captured on a light microscope (Nikon Eclipse E400, Nikon, Melville, NY). An iPhone 6 (Apple, Cupertino, CA) or Pixel 3 (Google, Mountain View, CA) smartphone was attached to the ocular lens of the light microscope using a NexYZ 3-axis universal smartphone adapter (Celestron, Torrance, CA). Liver tissue images were captured at 10x, 20x, and 40x across the entire slide specimen in nonoverlapping concentric circles. Deidentified images were downloaded in TIFF format into a central repository.

### Image Segmentation Algorithm Development

We applied an automated series of image processing techniques to each biopsy image to segment fat globules and quantify the ratio of fat to total liver area.

#### Conversion to Binary Color Format

**FIGURE 1.**
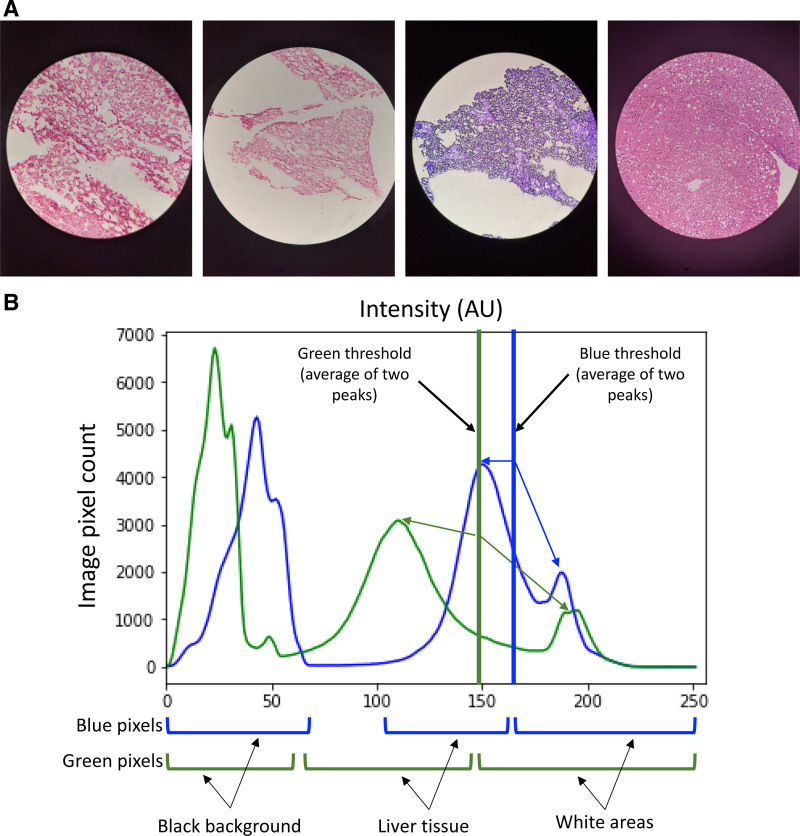
Representative cellphone-captured images of frozen liver biopsies. A, Biopsy images can vary significantly in appearance depending on the device used to capture the image, natural variation in the liver samples, and processing methods. B, To handle high variance in color across images, conversion from RGB to binary (black and white) was performed by calculating a unique RGB threshold for each image. For each individual image, pixels with blue or green intensity below the calculated threshold representing liver tissue (excluding black pixels representing the image border) are redefined as black pixels in the binary image. Pixels with blue or green intensity above the threshold representing white space such as steatosis were designated as white pixels in the binary image. RGB, red-green-blue.

Steatotic droplets, tissue disruption or tears, vascular lumen, and slide background do not stain positively with H&E and present as white or white-adjacent (Figure 1A). A black circular outline indicates the border of the smartphone image through the ocular lens of the microscope. These distinctions in color were used to convert the red-green-blue (RGB) smartphone images to binary format, where white pixels designate regions of possible steatosis and black pixels represent liver tissue.

Conversion from RGB to binary with minimal information loss is dependent on finding an ideal RGB threshold value: pixels with values above the threshold become white, whereas pixels with values below that threshold become black. To handle high variance in color across images, we calculated a unique threshold for each image by representing the RGB values of all pixels in an image as a histogram, then calculating the mean of the two highest peaks above an experimentally determined lower bound for green and blue values separately (red values had no effect) (Figure 1B).

#### Binary Image Enhancement

**FIGURE 2.**
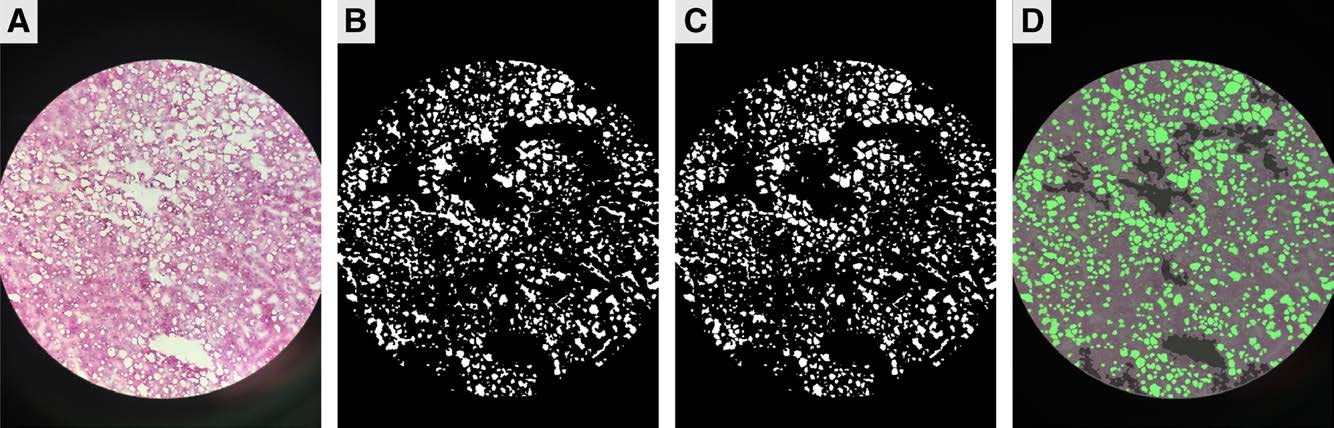
Representative image process techniques for identifying steatosis. A, Initial unprocessed image of frozen liver biopsy is shown. B, Converted raw image to binary format with morphological erosion. C, Binary image after watershed processing further separates steatotic droplets in proximity. D, Final image after fat segmentation with mask overlay demonstrating steatotic droplets in green.


After obtaining a binary image, morphological erosion was performed to reduce sparsely occurring noise by shrinking white regions and enlarging black regions. This step was necessary because tiny white or black regions may appear in the binary image depending on image quality. These regions are more likely to not represent steatosis but may mimic steatotic droplets. In addition, large nontissue background regions in the image were removed (Figure 2A and B). This was performed by setting a size value threshold of white regions to be greater than the largest fat droplet. The contour area (CA) was defined as the number of pixels in a contiguous white region of the image. The minimum enclosing circular area (MECA) was defined as the area of the smallest circle that encloses the contiguous white region. The circularity (C_1_) was defined as CA divided by MECA (Equation 1). For the 640 × 480 pixel histology images at 20x magnification, contiguous white regions with CA >2000 pixels, or with CA >600 pixels and circularity <0.3, were defined as nontissue background regions and filtered out. These threshold values were determined experimentally to be larger than any macrosteatotic droplet.

$$\begin{array}{l} C_{1}=\frac{CA}{MECA} \end{array}$$ (1)

In certain livers, steatotic droplets were tightly clustered to the extent that they were not visually distinguishable and presented instead as a unified white mass. These regions are prone to misinterpretation by the algorithm as histologic artifacts like tearing or vascular lumen. To address this issue, a watershed transformation was applied, which separated clustered fat regions into individual globules (Figure 2C).

#### Fat Segmentation

Area and circularity were used to determine which white regions in the binary image represented steatotic droplets. In images at 20x magnification, a region was potentially classified as a steatotic droplet if it had an area between 2 and 1999 pixels, inclusive, with a circularity above 0.2. The same definition for circularity was used as in Equation 1. Morphological dilation, a process that adds pixels to object boundaries, was then applied to restore the pixels of the fat regions that were previously removed during morphological erosion. The resulting image after dilation is the mask image, which contains regions of the original image that correspond to identified steatotic droplets (Figure 2D).

#### Additional Watershed and Filtering Step

An additional processing step was performed as some steatotic regions remained connected to nonsteatotic regions. The second watershed and filtering step allow separation of these connected regions and stricter filtering of nonsteatotic regions by using a higher circularity threshold. In this step, for 20x magnification images, a region was classified as a steatotic droplet if it had an area between 2 and 499 pixels, inclusive, with a circularity >0.7. The circularity *C*_*2*_ of a region was calculated using Equation 2, where *A* is the area of the region and *P* is the perimeter of the region.


$$\begin{array}{l} C_{2}=\frac{2\pi \sqrt{\frac{A}{\pi }}}{P} \end{array}$$ (2)

#### Calculation of Histologic Steatosis

Both the original unedited biopsy images and the final binary masked images for each individual liver were used to calculate a steatosis score. The sum of the number of white pixels in the mask image equates to the number of pixels of identified steatotic droplets. Because the size of the individual original images and the masked images are equal, the estimated percent steatosis for each image was calculated as the number of white pixels in the masked image divided by the number of liver tissue pixels in the original image. The final percent steatosis for each liver represents the mean of the steatosis estimates for all biopsy images for that individual liver, omitting outliers (±1 SD).

### Open Access Code Sharing

All software code along with examples of deidentified liver images are available at https://github.com/mit-quest/mgh-liver-segmentation.

### Statistical Analysis

Interobserver agreement between pathologists was measured using the intraclass correlation (ICC) with a 2-way mixed-effects model. ICC is a statistical measure of rater agreement on the same targets. An ICC <0.5 indicates poor, 0.5 to 0.75 moderate, 0.75 to 0.9 good, and >0.9 excellent agreement. Correlation between pathologists and algorithm estimates was measured using Pearson’s correlation coefficient and linear regression. Stata 15.1 (StataCorp, College Station, TX) and Prism 9 (GraphPad Software, San Diego, CA) were used for statistical analysis and visualization. The threshold for significance was set to *P* < 0.05.

## RESULTS

### Interobserver Agreement of Pathologists

**FIGURE 3.**
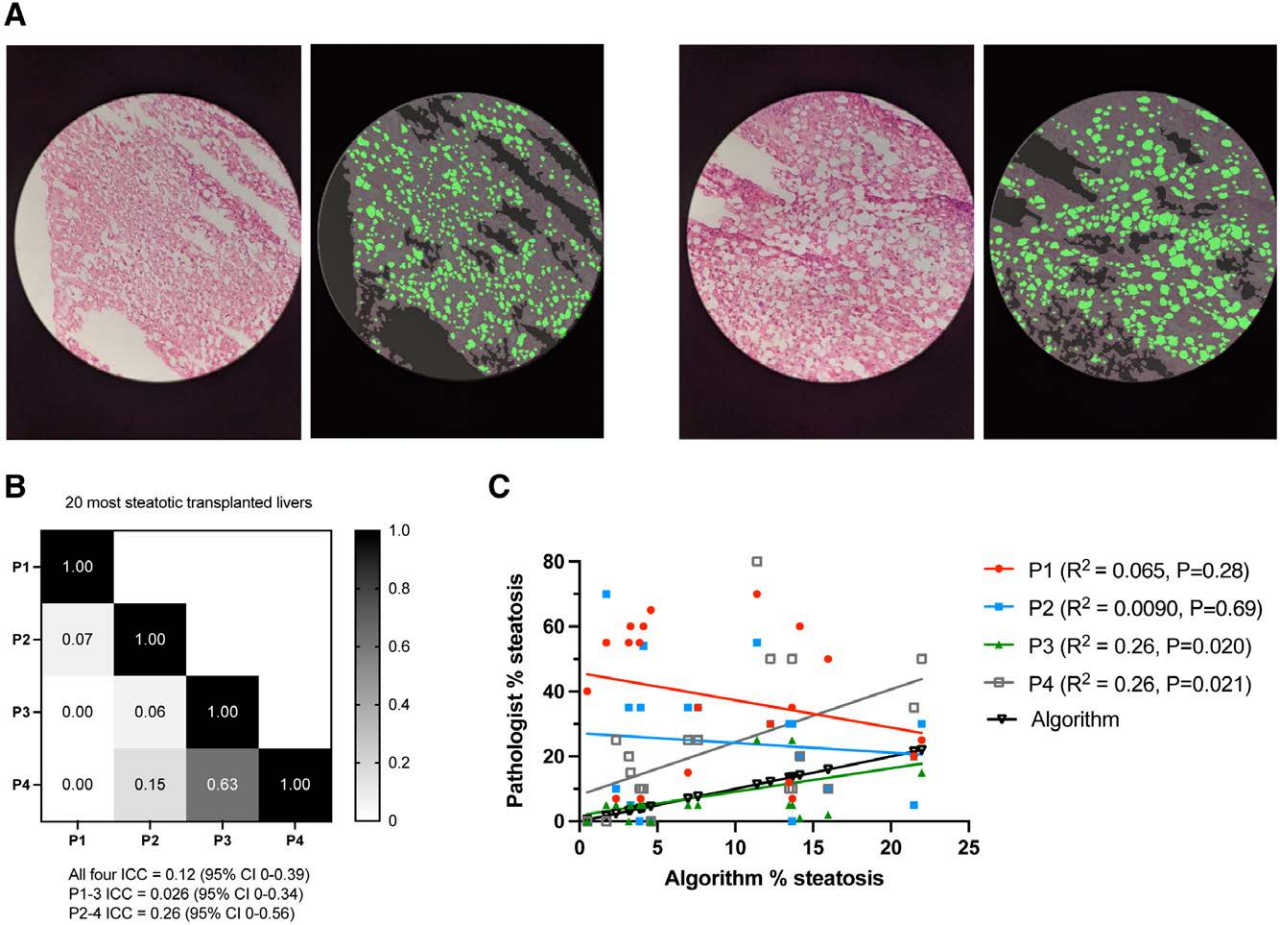
Wide variation in pathologist and algorithm interpretation of clinical biopsy samples. A, Two representative raw and processed images from the same liver biopsy are shown. Four pathologist macrosteatosis scores of the image slide were 70%, 55%, 25%, and 80%. The algorithm estimate was 11.4%. B, Heatmap of Pearson correlation between individual pathologists for 20 clinical biopsy slides with the greatest steatosis. ICC scores among various pathologist cohorts are provided. C, Correlation of individual pathologist steatosis estimates with algorithm estimates. The 2 most experienced pathologists (P3 and P4) had significant score correlation with algorithm estimates. ICC, intraclass correlation; P, pathologist.

Intraclass correlation was performed to measure interobserver (pathologist) agreement of liver steatosis scores. ICC between 3 pathologists of diverse training backgrounds (P1–3) for all 91 imaged frozen section slides was 0.20 (95% CI, 0.074-0.34), indicating poor agreement. However, the majority of livers included had minimal (<5%) or no steatosis (0%). From a clinical perspective, pathologists had a significant disagreement on 28 of 91 (30.8%) livers, indicating discrepant scores above and below the presumed 30% steatosis threshold for transplant. In practice, almost one third of these transplanted livers could have been discarded as a result, depending on which pathologist’s score was used. When the analysis was restricted to the 20 grafts with the most apparent steatosis, agreement worsened (ICC 0.026; 95% CI, 0-0.34). Replacing the least experienced pathologist (P1) with an expert liver pathologist (P4) only marginally improved interobserver agreement (ICC 0.26; 95% CI, 0-0.56) (Figure 3A and B). When the steatosis estimates of pathologists P1 through 4 for the 20 livers with the most apparent steatosis were correlated with the algorithm’s estimates, the estimates of the 2 most experienced pathologists demonstrated statistically significant but relatively weak correlations (R^2^ = 0.26, *P* < 0.05 for both) (Figure 3C). From a clinical perspective, only 3 of 20 livers (15%) had no disagreement between scores, indicating that 17 of 20 (85%) livers could have been discarded depending on which pathologist’s score was used for decision making.

### Correlation of Algorithm Estimates with Standardized Slide Estimates

**FIGURE 4.**
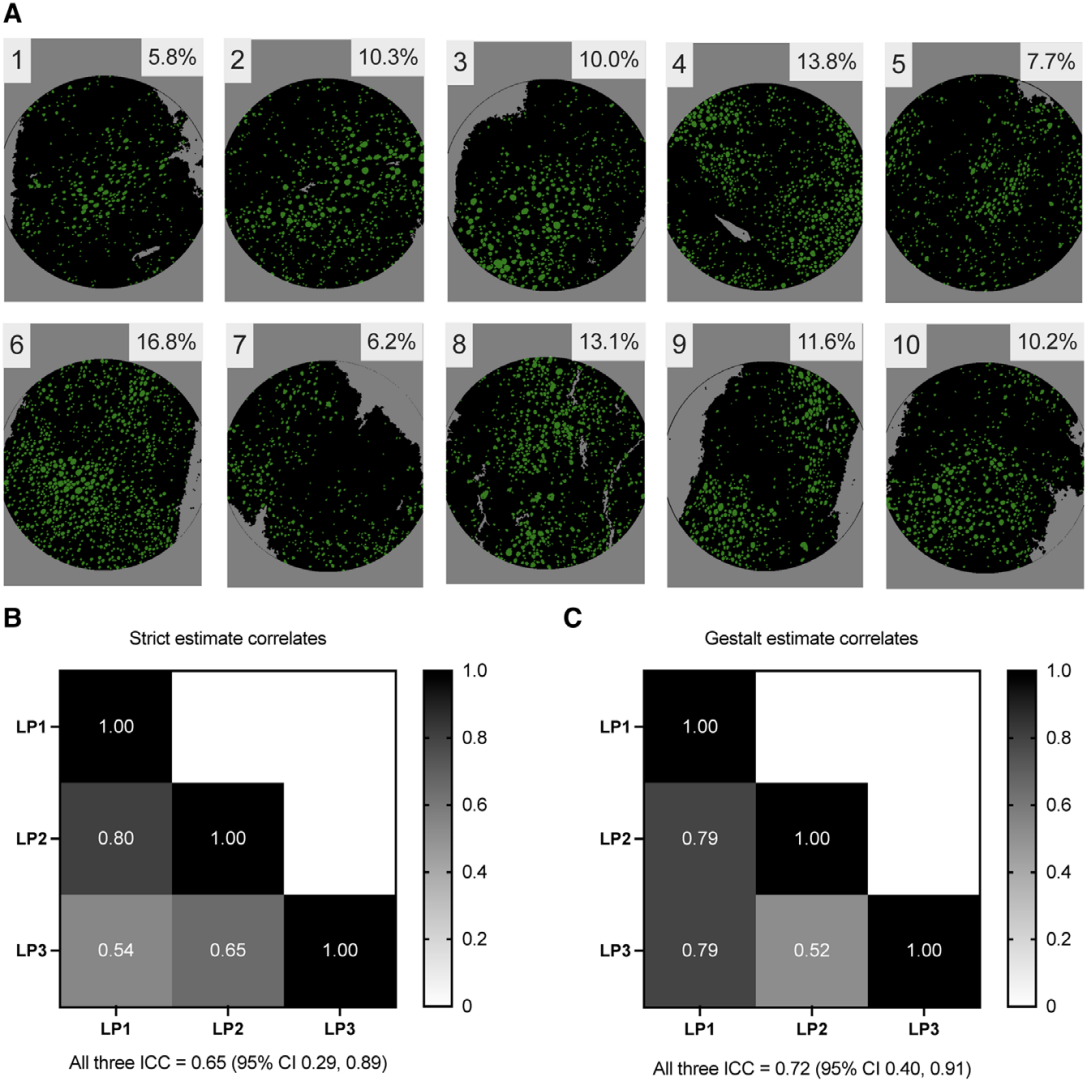
Manually generated examples of steatosis and pathologist agreement. A, Ten images were manually generated to mirror examples of liver biopsies with steatosis. Fat droplets are shown in green and liver tissue in black. The algorithm estimate of percentage steatosis is shown in the top right corner of each image. Heatmaps of ICC among expert liver pathologists when providing (B) strict vs (C) gestalt estimates of steatosis for the 10 images in (A). LP, liver pathologist; ICC, intraclass correlation.


Given the remarkable discrepancy among pathologists’ estimates of liver steatosis, an additional set of control images was created to evaluate the inherent variability in interobserver agreement. Ten control images were created with fat segmentation based on liver biopsies, where regions representing fat are colored green, regions representing liver tissue are colored black, and regions representing neither liver tissue nor fat are colored gray (Figure 4A). In this binary mode, the calculation purely counts green and black pixels, so the algorithm’s estimate of the steatosis content serves as the “ground truth.” For each image, 3 expert liver pathologists (LP 1–3) provided a strict estimate and a gestalt estimate of fat content, considering each image independently. For the strict estimate, pathologists were instructed to use Equation 3:

$$\begin{array}{l} Strict\;estimate=\;\frac{Sum\;of\;green\;\;areas\;\;\;}{Sum\;of\;\;green\;areas\;+\;\;Sum\;\;of\;\;black\;areas} \end{array}$$ (3)

**FIGURE 5.**
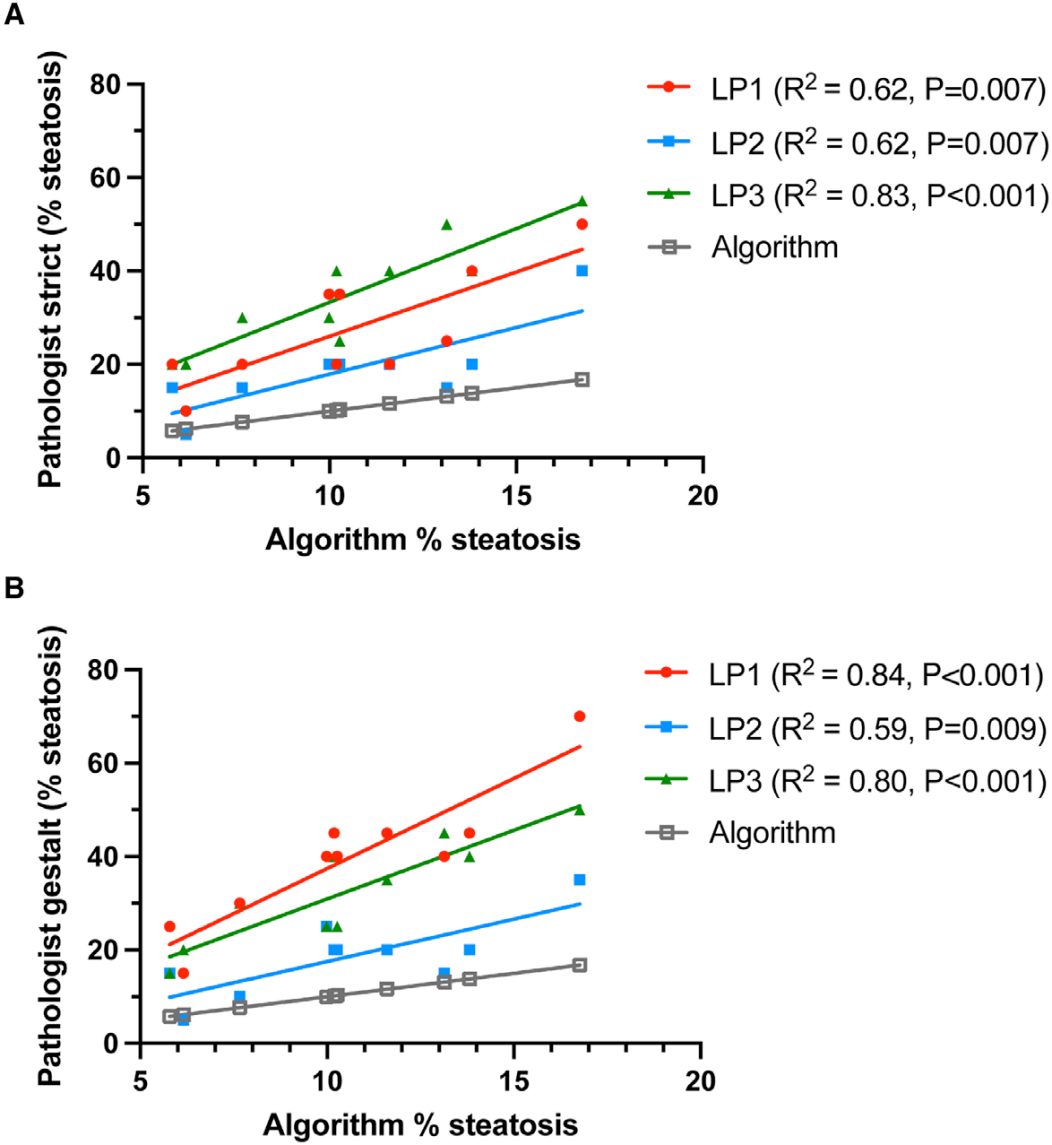
Expert liver pathologist estimate correlations with algorithm estimates. Graphs demonstrating correlation between (A) strict and (B) gestalt estimates of 3 expert LPs with algorithm estimates of manually generated images from Figure 4A. LP, liver pathologist.

For the gestalt estimate, the pathologist considered not only the factors in the strict estimate but also the extent and distribution of the liver tissue in an image affected by fat, which more closely approximates the assessment of steatosis in practice. Interobserver agreement was moderate in this setting for strict (ICC 0.65; 95% CI, 0.29-0.89) and gestalt estimates (ICC 0.72; 95% CI, 0.40-0.91) (Figure 4B and C). However, when compared with the algorithm’s ground truth calculations, the pathologists’ strict and gestalt estimates of percentage macrosteatosis were categorically higher (Table 1). Figure 5 demonstrates the linear relationship between pathologist and algorithm steatosis estimates. It is worth noting that each pathologist has their own slope, or factor, by which their estimate can be correlated with the algorithm. This indicates a tendency for some pathologists to score higher at the same rate and others to score lower.

**TABLE 1.** Expert LP strict and gestalt estimates of binary control steatosis images

Image	Algorithm Estimate	LP 1 strict estimate	LP 1 gestalt estimate	LP 2 strict estimate	LP 2 gestalt estimate	LP 3 strict estimate	LP 3 gestalt estimate
1	5.8	20	25	15	15	20	15
2	10.3	35	40	20	20	25	25
3	10.0	35	40	20	25	30	25
4	13.8	40	45	20	20	40	40
5	7.7	20	30	15	10	30	30
6	16.8	50	70	40	35	55	50
7	6.2	10	15	5	5	20	20
8	13.1	25	40	15	15	50	45
9	11.6	20	45	20	20	40	35
10	10.2	20	45	20	20	40	40

LP, liver pathologist.

Each cell is conditionally shaded to represent distance between algorithm’s pixel-based calculation and the pathologist’s estimate. Darker shading indicates larger divergence.

## DISCUSSION

In this study, we created a non-ML digital vision algorithm using only smartphone-captured images of liver biopsy histology. This unique approach mimics the real-world practice of obtaining frozen sections of liver biopsies at the time of organ procurement. This would allow the procurement surgeon to image the frozen section using a smartphone and standard light microscope to obtain an estimate of liver steatosis instantaneously. In addition, our algorithm’s steatosis estimates correlate strongly with those of expert liver pathologists.

In creating the vision-based algorithm, we were able to avoid the subjective pitfalls of steatosis scoring. Primarily, this is the result of differing interpretations of steatosis as the percentage of the available tissue biopsy. This is vividly apparent in the recently published Banff consensus study for assessment of donor liver steatosis.^22^ Even among this group of expert liver pathologists, there was notable disagreement on steatosis scores in livers with more than minimal (>5%) steatosis. Moreover, discordant expert scores were demonstrated to negatively affect graft utilization rates because overestimation leads to graft underutilization. This further highlights a significant real-world problem leading to high rates of graft discard. Donor procurement is often performed at local institutions without subspecialty pathologists to interpret frozen section liver biopsies, resulting in wide variability in assessment. This is similarly borne out in this study, where ICC was quite poor when pathologists of varying experience were asked to score the same set of liver slides. Automated steatosis scoring at the point-of-care would greatly impact the ability to make accurate estimates of steatosis and is likely to improve graft utilization rates.

Similar progress has been achieved with the use of ML algorithms. Several studies have applied ML for the assessment of fibrosis and inflammation in livers from patients with nonalcoholic fatty liver disease and nonalcoholic steatohepatitis.^18,20^ Other groups have also applied deep learning to accurately score steatosis in a cohort of donor liver biopsies.^17,21,23,24^ However, these published studies often use whole-slide images or formalin-fixed paraffin-embedded tissue as the basis for algorithm testing and validation, which makes the application of these technologies less viable to real-world situations. Often, whole-slide imaging is not available at the time and location of organ procurement, and it is rarely feasible to return to the recipient hospital for further imaging analysis. In this respect, the vision algorithm reported here avoids these limitations by using relatively low-resolution smartphone-captured images of frozen biopsies as the input for analysis, allowing the procuring surgeon to make timely decisions. Additionally, this algorithm circumvents a limitation faced by many ML systems in obtaining a sufficiently diverse dataset for training. It can therefore be used to generate training data at scale for ML models to further refine the segmentation, either by incorporating its output directly or as a starting point to facilitate manual labeling using the segmented image output along with a steatosis score that the algorithm produces.

An additional finding of significance was that pixel-based steatosis scoring resulted in lower absolute steatosis scores, though there was a linear correlation with pathologist estimates. This finding demonstrates the difference between how the human brain ascribes a visual estimate of fractions compared with the absolute pixel count of a computer. Another contributor may be that pathologists tend to characterize steatosis by the percentage of hepatocytes containing lipid droplets and not by the overall percentage of the slide having steatosis.^25^ To best account for this, the algorithm removed sinusoidal blank space, vascular lumens, and processing artifacts from the calculation of total liver tissue. In practice, this finding indicates that 30% steatosis looks different to one pathologist compared with another. Moreover, what is considered 30% steatosis by a pathologist may translate to a lower percentage assessment by the algorithm. The human to algorithm difference is noteworthy because percentage steatosis thresholds for graft utilization may change if the algorithm’s score is used. Clinicians who intend to use the digital algorithm must be aware of this discrepancy and incorporate it into their decision-making process. Alternatively, the algorithm can be calibrated to output an adjusted score based on an individual institution’s cohort of pathologist estimates using the “ground truth” images (Figure 4A). This linear score discrepancy is a limitation of creating a vision-based algorithm without pathologist biopsy annotations. A recently published study by Narayan et al using an ML-based vision algorithm similarly showed a large discrepancy between algorithm steatosis scores and pathologist scores (median 3% versus 20%, respectively, *P* < 0.001). Notably, the ML algorithm scores distinguished grafts at risk of developing early allograft dysfunction.^26^

Other limitations include the combination of both microsteatotic and macrosteatotic droplets in the algorithm steatosis assessment. Distinguishing between the 2 would provide a more accurate representation of a graft’s quality and better help the surgeon in determining suitability for transplant because microsteatosis has less of an impact on liver transplantation outcomes.^27^ Future studies should incorporate prospective analysis of donor biopsy slides by both the digital algorithm and a liver pathologist to better understand score discrepancies and its implications for clinical decision making. Furthermore, we were unable to correlate steatosis scores with clinical outcome parameters, such as early allograft dysfunction, as most of the available archived liver biopsy slides predated the transition to our current electronic medical record system. These analyses in subsequent studies would provide insight into the utility of the digital algorithm in graft selection. In addition, the accuracy of the algorithm assessment was unable to be rigorously validated, though the strong linear relationship with experienced liver pathologist scores is supportive. Future work could include manual comparison of the algorithm’s mask overlays with slides annotated by pathologists or oil-specific staining of biopsy slides for comparison. In our study, additional tissue samples for the archived slides were not available for reprocessing.

To conclude, this study demonstrates proof of the concept that smartphone-captured images can be used in conjunction with a digital algorithm to measure steatosis. Additional software development and prospective studies are needed to allow its use at the point-of-care in clinical transplantation.

## ACKNOWLEDGMENTS

The authors would like to thank the patients, donors, and their families for making this work possible. K.F. would like to thank Kuan Wei Huang for his contributions to the algorithm for formalin-preserved samples.
